# Copy number alterations in metastatic and early breast tumours: prognostic and acquired biomarkers of resistance to CDK4/6 inhibitors

**DOI:** 10.1038/s41416-024-02804-6

**Published:** 2024-08-02

**Authors:** Marie-Paule Sablin, Pierre Gestraud, Sarah Flora Jonas, Constance Lamy, Magali Lacroix-Triki, Thomas Bachelot, Thomas Filleron, Ludovic Lacroix, Alicia Tran-Dien, Pascal Jézéquel, Marjorie Mauduit, Janice Barros Monteiro, Marta Jimenez, Stefan Michiels, Valery Attignon, Isabelle Soubeyran, Keltouma Driouch, Nicolas Servant, Christophe Le Tourneau, Maud Kamal, Fabrice André, Ivan Bièche

**Affiliations:** 1 Department of Drug Development and Innovation (D3i), Institut Curie, Paris, France; 2 Bioinformatics and Computational Systems Biology of Cancer, PSL Research University, Mines Paris Tech, INSERM U900, Paris, France; 3 Service de Biostatistique et d’Épidémiologie, Gustave Roussy, Université Paris-Saclay, Villejuif, France; 4 Oncostat U1018, Inserm, Université Paris-Saclay, Équipe Labellisée Ligue Contre le Cancer, Villejuif, France; 5 Department of Pathology, Gustave Roussy, Villejuif, France; 6 Department of Medical Oncology, Centre Léon Bérard, Lyon, France; 7 Department of Biostatistics, Institut Claudius-Regaud, IUCT Oncopole, Toulouse, France; 8 Department of Medical Biology and Pathology, Gustave Roussy, Villejuif, France; 9 Genomic Platform and Biobank, CNRS UMS3655-INSERM US23, AMMICA, Gustave Roussy, Villejuif, France; 10 Bioinformatics Platform, Gustave Roussy, Villejuif, France; 11 Omics Data Science Unit, Institut de Cancérologie de l’Ouest (ICO), Angers-Nantes, France; 12 Precision Medicine Group, UNICANCER, Paris, France; 13 R&D Unicancer, Paris, France; 14 Cancer Genomic Platform, Centre Léon Bérard, Lyon, France; 15 Department of Biopathology, Institut Bergonié, Bordeaux, France; 16 Department of Genetics, Institut Curie, Paris University, Paris, France; 17 Department of Medical Oncology, Gustave Roussy, Villejuif, France

**Keywords:** Breast cancer, Metastasis

## Abstract

**Background:**

Copy number alterations (CNA) are acquired during the evolution of cancers from their early stage to metastatic stage. This study aims at analysing the clinical value of the identified metastasis-associated CNAs both in metastatic breast cancers (mBCs) and early breast cancers (eBCs).

**Methods:**

Single-nucleotide polymorphism (SNP)-array was performed on 926 biopsies from mBC patients, enrolled in SAFIR02-BREAST prospective trial. CNA profiles of eBCs from The Cancer Genome Atlas Breast Invasive Carcinoma (*n* = 770), Molecular Taxonomy of Breast Cancer International Consortium (*n* = 1620) and PACS04 trial (*n* = 243) cohorts were used as references for comparing mBCs and eBCs CNA profiles. Overall survival was the considered survival endpoint.

**Results:**

Among the twenty-one genes frequently altered in ER + /HER2− mBCs: focal amplification of *TERT* was associated with poor outcome in the ER + /HER2− mBC population. Among the ER + /HER2− mBCs patients for whom CDK4/6 inhibitors information before biopsies collection was available: we identified seven genes on post-treatment biopsies, including the *cyclin-dependent kinase 4* (*CDK4)*, which was amplified in 9.8% of the ER + /HER2− mBCs pretreated population, as compared to 1.5% in the ER + /HER2− mBCs unpretreated population (*P* = 2.82E-04) as well as the 3 eBC populations. *CDK4* amplification was associated with poor outcome in the ER + /HER2− eBCs.

**Conclusions:**

This study provides insights into the biology of mBCs and identifies clinically useful genomic features for future improvement of breast cancer patient management.

## Introduction

Breast cancer is the most common malignancy among women worldwide [1]. In western countries, more than 95% of breast cancers are diagnosed at the early stage. While the incidence of distant relapse is decreasing, 20–30% of patients with early breast cancer (eBCs) still die from metastatic disease [2]. Over the last two decades, significant advances have been made in the management of breast cancer. Breast tumours are diverse in their natural history and their responsiveness to treatments. Indeed, breast cancer is considered as a heterogeneous disease at a morphologic, immunohistochemical (IHC), and even molecular level [3]. A better understanding of the biology has led to overcome the historical classification in three subgroups hormone receptor-positive (HR +), human epidermal growth factor receptor 2 overexpressed or amplified (HER2 +) and triple-negative breast cancer (TNBC), defined by the lack of HRs and HER2 overexpression or amplification. Back in 2000, Sorlie et al. individualised five intrinsic molecular subtypes using gene expression profiling: luminal A, luminal B, HER2 enriched, basal-like and normal breast-like [4]. Recently, Thennavan et al. described no less than 12 subtypes of breast cancer [5]. Besides this better characterisation, new therapeutic options have enriched the available therapeutic arsenal, such as antibody drug conjugated and cyclin-dependent kinase 4 and 6 (CDK4/6) inhibitors. Since 2018, CDK4/6 inhibitors (CDK4/6i) (e.g., palbociclib, ribociclib or abemaciclib) combined to endocrine therapy became the backbone of the treatment of HR + /HER2− breast cancer [6]. They improve the outcome of patients in the first and second line in the metastatic setting. Moreover, one of them, abemaciclib, has demonstrated benefits for patients with high risk at an early stage [7].

Despite all the advances, treatment choices are still based on the historical three IHC subtypes and no biomarkers are available to guide the management of our patients. Moreover, this broad classification does not take into account the significant tumour evolution during metastatic progression which is more driven by selective pressures like alterations occurring in tumour suppressor genes (TSG) and oncogenes [8, 9].

Due to tumour evolution and treatment pressure, the genomic alterations in metastatic breast cancer (mBC) seem to differ substantially from the primary tumour [10–13]. To date, in the era of precision oncology, few large-scale genomic studies focused on mBCs to identify new clinical biomarkers [14–16].

The genomic landscape of mBCs is key to identify new prognostic and predictive biomarkers including genes and pathways involved in drug resistance and metastatic processes to generate new strategies to treat these patients. Genetic alterations found to be more frequently altered in mBCs could pre-exist in the early eBCs as subclones of cancer cells and be potential biomarkers of prognostic and predictive values in the eBCs.

We have previously compared the efficacy of maintenance treatment with a targeted therapy matched to genomic alteration in 238 HER2− mBC patients randomised in two trials (NCT02299999 and NCT03386162). These patients were previously enrolled in the SAFIR02-BREAST trial (NCT02299999) [17]. Genes more frequently amplified or deleted in the SAFIR02 mBC patients (*n* = 926) as compared to eBC cohort data patients were reported. We have described 19 genes that were frequently altered in our series of ER + /HER2− mBC in comparison with the ER + /HER2− eBC.

In this study, we would like to investigate the prognostic and predictive clinical interest of these metastasis-associated genes in mBC and eBC populations in both the ER + /HER2− and TNBC sub-populations.

## Methods

### Patients and breast cancer datasets

**Fig. 1:**
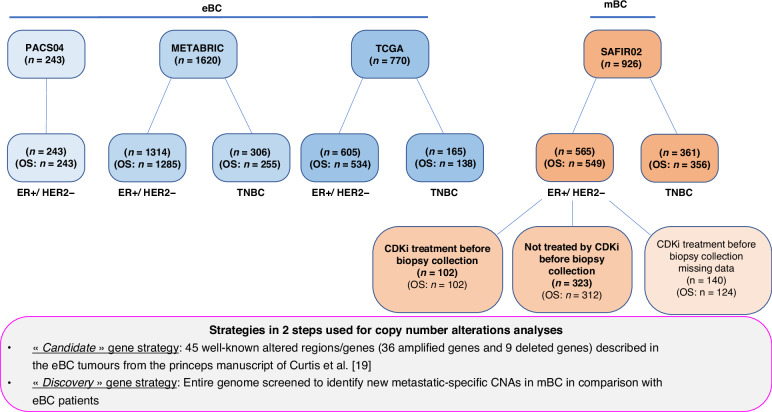
Study design. Genes frequently altered in mBC and eBC populations in the ER + /HER2− or TNBC sub-populations.

The study workflow is reported in Fig. 1. Patients eligible for this study were selected from the SAFIR02-BREAST prospective trial (NCT02299999, *n* = 926) for the mBC patients, and from The Cancer Genome Atlas Breast Invasive Carcinoma (TCGA, *n* = 770), the Molecular Taxonomy of Breast Cancer International Consortium (METABRIC, *n* = 1620) and the PACS04 trials (*n* = 243) for the eBC patients. All patients are female.

### SAFIR02-BREAST mBC patients

The mBC population included 926 patients, who underwent a biopsy of a metastatic site between April 2014 and September 2019 in the context of the prospective, not blinded, randomised, phase II trial SAFIR02-BREAST (NCT02299999), and for whom the genomic profile was available (Supplementary Table S1). The inclusion criteria and ethics approval for SAFIR02-BREAST were described by André et al. [17].Among the 926 mBC tumours, overall survival (OS) studies have been performed on 356 TNBC patients (39%) and 549 ER + /HER2− breast cancer (61%). The oestrogen receptor (ER) and HER2 status were determined on the primary tumour sample.Among the 56 5 ER + /HER2− mBC patients, we had the information on the CDK4/6i treatment before biopsies collection for 425 patients: 323 were unpretreated with CDK4/6i whereas 102 received the CDK4/6i (Fig. 1). For this patient population analyses were performed on the post-treatment biopsies.

### eBC tumour datasets

Genomic and clinical data from TCGA, METABRIC and PACS04 studies were used in this study (Supplementary Table S2). TCGA data were downloaded from Genomic Data Commons and METABRIC data from the European Genome-Phenome Archive. PACS04 trial data were obtained from the study sponsor, UNICANCER. Copy number analysis was performed in the same way for the eBC and the mBC tumours (as described below). Among the 2633 eBC tumours from TCGA, METABRIC and PACS04, OS studies have been performed on 393 TNBC patients (16%) patients and 2062 ER + /HER2− breast cancer (84%).

### SNP-array in the SAFIR02-Breast trial (mBC tumours)

Patients across France had access to molecular testing in five regional molecular cancer genetics platforms labelled by UNICANCER for the SAFIR02-Breast trial. The tumour samples were qualified for further genomic testing if more than 30% of the cells in the biopsy sample were cancer cells for frozen core biopsies and for formalin-fixed paraffin-embedded tumour biopsies.

DNA was isolated from six FFPE tissue sections (each 6-μm thick). A seventh tissue section was stained with H&E. The tumour-rich areas were macro-dissected using a single-use blade, and the samples underwent proteinase K digestion in a rotating incubator at 56 °C for 3 days. DNA was extracted using the Nucleospin 8 Tissue kit (Macherey-Nagel). Isolation of DNA from frozen core biopsies was performed using the AllPrep DNA/RNA Mini kit (Qiagen) according to the manufacturer’s protocol. DNA was quantified using a Qubit 2.0 Fluorometer (Quant-iT dsDNA BR Assay Kit; Thermo Fisher Scientific), according to the manufacturer’s instructions [18].

### Determination of focal amplifications and homozygous deletions by bioinformatics analysis in the four BC cohorts (mBC and eBC tumours)

Copy number alterations were detected prospectively using the CytoScan HD Array Kit (Affymetrix, a Thermo Fisher Scientific company) for the fresh-frozen tissues and the OncoScan FFPE Assay Kit (Affymetrix) for the FFPE tissues. The OncoScan FFPE Assay Kit is a microarray designed specifically for use with degraded DNA, as is found in FFPE tissue. Both the OncoScan and the CytoScan arrays used single-nucleotide polymorphism probes to provide DNA copy number variations, according to the manufacturer’s instructions. Copy number alterations (CNAs) from CytoScan and OncoScan were defined using the R package EaCoN (v0.3.3), available at https://github.com/gustaveroussy/EaCoN/. Briefly, log2 relative ratios was calculated, a centralisation of the profile set the baseline (two copies being the neutral level) from which CNAs were estimated. Break points in the log2 relative ratio continuity were identified by segmenting the profile. Among the CNAs, only the focal amplifications and the homozygous deletions were considered. Focally amplified genes were defined as genes fully included in a DNA segment smaller than 10 Mb and with a copy number greater or equal to 6. Homozygous deleted genes were defined as genes fully or partially included in a DNA segment with a copy number equal to 0. For each focal amplified region, we identified gene(s) located in the smallest common focal amplified DNA segment (SCFADS). If several genes were located in this SCFADS, we have indicated the gene(s) from the OncoKB Cancer Gene List (https://www.oncokb.org/cancerGenes) [18].

### Candidate and discovery strategies used for CNAs analyses from ER + /HER2− and TNBC patients

Briefly, as a first intention, we used a “candidate strategy” by testing the 45 well-known altered regions/genes (36 amplified genes and 9 deleted genes) described in the eBC tumours from the princeps manuscript of Curtis et al. [19], and as a second intention, we used a “discovery strategy” by screening the entire genome to identify new metastatic-specific CNAs in mBC patients in comparison with eBC patients.

### Real-time RT-PCR

Quantitative values were obtained from the cycle number (Ct value) at which the increase in the fluorescence signal associated with the exponential growth of PCR products started to be detected by the laser detector of the ABI Prism 7900 sequence detection system (Perkin-Elmer Applied Biosystems, Foster City, CA), using PE biosystems analysis software according to the manufacturer’s manuals.

The precise amount of total RNA added to each reaction mix (based on optical density) and its quality (i.e., lack of extensive degradation) are both difficult to assess. Therefore, transcripts of the *TBP* gene (Genbank accession NM_003194) encoding the TATA box-binding protein (a component of the DNA-binding protein complex TFIID) were also quantified as an endogenous RNA control. Each sample was normalised on the basis of its *TBP* content. Results expressed as N-fold differences in *CDK4* gene expression relative to the *TBP* gene and termed “N*cdk4*”, were determined as N*cdk4* = 2^ΔCtsample^, where the ΔCt value of the sample was determined by subtracting the average Ct value of *CDK4* gene from the average Ct value of *TBP* gene.

The nucleotide sequences of the primers used for PCR amplification were the following: CDK4-U (5’-GCCCAGTGCAGTCGGTGGTA-3’) and ER + CDK4-L (5’-GGTTAAAAGTCAGCATTTCCAGCAG-3’) and TBP-U (5’-TGCACAGGAGCCAAGAGTGAA-3’) and TBP-L (5’-CACATCACAGCTCCCCACCA-3’). PCR was performed using the SYBRs Green PCR Core Reagents kit (Thermo Fisher Scientific). To avoid amplification of contaminated genomic DNA, one of the two primers was placed at the junction between two exons.

The conditions of RNA extraction, cDNA synthesis and PCR were as previously described [20].

### Statistical analyses

Data were summarised according to frequency and percentage for qualitative variables. Comparisons between groups were assessed using the chi-square or Fisher’s exact test for qualitative variables.

OS was the considered survival endpoint. It was measured as the time from inclusion to death from any cause. Patients alive were censored at their last follow-up. Patients without this information were excluded from the survival analysis. Multivariable analyses were carried out using Cox proportional hazards model. The specific contribution to the survival of each genetic alteration was studied, using a likelihood test, by comparing Cox models adjusted to the clinicopathological factors known in mBC and eBC with and without the considered genetic alteration. We applied a Benjamini–Hochberg correction for multiple tests. All statistical tests were two-sided and a *P* < 0.05 was considered statistically significant. Survival estimates were obtained with the Kaplan–Meier method and compared with the log-rank method. All statistical analyses were carried out using R v4.2 software.

## Results

### Patient characteristics

A total of 3559 patients included in four different cohorts were analysed: 926 mBC tumours and 2633 eBC tumours. The study workflow is reported in Fig. 1, and patient characteristics are reported in Supplementary Tables S1 and S2. Among the 926 mBC tumours (SAFIR02-BREAST trial), 361 (39.0%) patients had TNBC, and 565 (61.0%) had ER + /HER2− breast cancer (Supplementary Table S1). Among the 2633 eBC tumours from TCGA, METABRIC and PACS04, 471 (17.9%) patients had TNBC and 2162 (82.1%) had ER + /HER2− breast cancer (Supplementary Table S2).

### The CNA landscape in mBCs and eBCs

To reduce the complexity of CNA landscape, in this study, we specifically focused our approach on the detection of focal amplification and homozygous deletion events, as described in the method section. This approach allowed to highlight genes of clinical interest.

To be noted that we decided to study separately the ER + /HER2− and the TNBC mBC populations since the CNA profiles of these two populations are very different [19].

In ER + /HER2− mBC population, the focal amplification of *CCND1* (30.1%) and the homozygous deletions of *CDKN2A* (6.4%) dominated the CNA landscape. In ER + /HER2− eBC population, while amplification alterations of *CCND1* (14.1%) were also highlighted, homozygous deletion of *MAP2K4* (2.7%) was dominant in the CNA profile (Supplementary Fig. S1A).

In the metastatic triple-negative breast cancer (mTNBC) population, we observed a focal amplification of *MYC* (16.3%) and a homozygous deletion of *PTEN* (9.7%) as the more common CNA events. In the CNA landscape of early triple-negative breast cancer (eTNBC) population, *TRIM46* (10.8%) dominates as a focal amplification alteration, while *PTEN* (5.4%) dominates as a homozygous deletion (Supplementary Fig. S1B).

These four genes, *CCND1, CDKN2A, MYC* and *PTEN* identified as frequently altered in mBC populations are well-known driver genes in the eBC tumorigenesis [21].

### CNAs enriched in ER + /HER2− mBCs

**Fig. 2:**
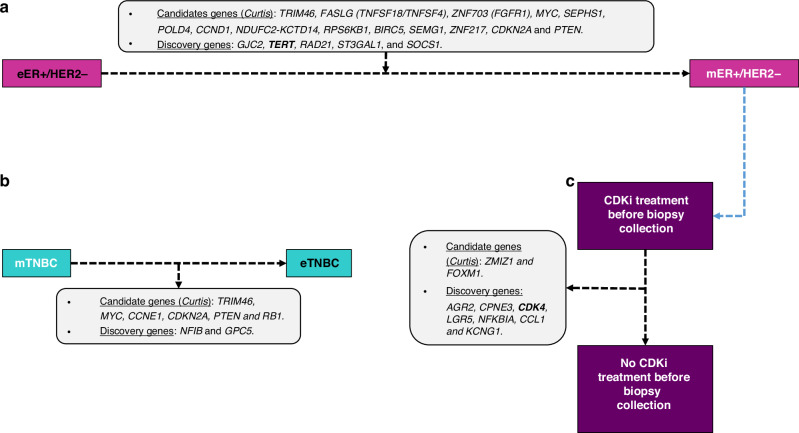
Genes frequently altered in mBC and eBC populations in the ER + /HER2- or TNBC sub-populations. **a** ER + /HER2− mBCs genes frequently altered in comparison to ER + /HER2− eBCs. **b** mTNBC genes frequently altered in comparison to eTNBC. **c** ER + /HER2- mBCs CDKi treatment before biopsies collection genes frequently altered in comparison to ER + /HER2− mBCs CDKi untreated before samples collection.

We previously used a “two steps” methodology for the ER + /HER2− BC populations (Figs. 1 and  2a) [17]. Briefly, as a first intention, we used a “candidate strategy” by testing the 45 well-known altered regions/genes (36 amplified genes and 9 deleted genes) described in the eBC tumours from the princeps manuscript of Curtis et al. [19], and as a second intention, we used a “discovery strategy” by screening the entire genome to identify new metastatic-specific CNAs in ER + /HER2− mBCs. We selected only the genes that were significantly more frequently altered in ER + /HER2− mBCs as compared to the three ER + /HER2− eBCs cohorts separately.

Among 45 well-known altered regions/genes, reported by Curtis [19], we previously identified 14 genes more frequently altered in ER + /HER2− mBCs as compared to the three ER + /HER2− eBC cohorts (Fig. 2a, Supplementary Fig. S1A and Supplementary Table S3) [17]. When the entire genome was analysed, 5 new genes were found to be more frequently amplified in ER + /HER2− mBCs (*GJC2, TERT, RAD21, ST3GAL1* and *SOCS1*).

### Prognostic interests of CNAs enriched in ER + /HER2− mBCs and ER + /HER2− eBCs

**Fig. 3:**
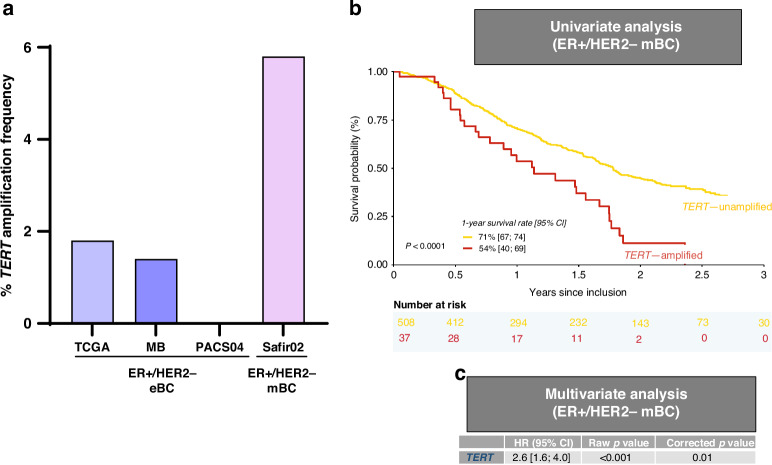
*TERT* amplification analysis in ER + /HER2− eBC and ER + /HER2− mBC. **a** *TERT* amplification frequency (%). **b** Overall survival in univariable analysis in ER + /HER2− mBC. **c** Overall survival in multivariable analysis in ER + /HER2− mBC.

Results of the univariable and multivariable analyses in mBC of the 19 CNAs enriched in 549 ER + /HER2− mBCs are reported in Supplementary Table S4. Among these 19 genetic alterations tested, focal amplifications of *TERT, RAD21* and *MYC* were significantly associated with poor OS both in univariable and multivariable analysis in the ER + /HER2− mBC (Supplementary Table S4). However, only amplification of *TERT*, which were more frequently amplified in ER + /HER2− mBCs (Fig. 3a) reach significance in univariate (corrected *P* < 0.0001, Fig. 3b) and multivariable analysis after correction for multiple testing (*P* = 0.01, Fig. 3c).

We hypothesised that these 19 genetic alterations found to be more frequently present in mBCs could pre-exist in the eBCs as subclones of cancer cells and be potential biomarkers of poor prognosis in the eBCs. In consequence, we analysed the link between these 19 genetic alterations and the outcome of the 2062 ER + /HER2− eBC patients (all eBC tumours from the TCGA, METABRIC and PACS04 cohorts). Results of the univariable and multivariable analyses are reported in Supplementary Table S5. Among the 19 genetic alterations tested, focal amplifications of *RPS6KB1, SEMG1, GJC2* and *SOCS1*, and homozygous deletions of *CDKN2A* and *PTEN* were significantly associated with poor OS in multivariable analysis. It should be noted that these 6 genes did not reach significance after correction for multiple testing.

### CNAs enriched in mTNBCs, and prognostic interests in mTNBCs and eTNBCs

Well-known altered genes and “discovery strategy” genes were analysed as previously described in the ER + /HER2− cohorts to identify genes that were more frequently altered in the 361 mTNBCs as compared to 471 eTNBC (only TCGA, and METABRIC cohorts because the PACS04 cohort is exclusively composed by ER + /HER2− eBCs).

Among the 45 well-known altered regions/genes, no gene was found to be more frequently altered in mTNBC compared to the eTNBC cohorts (Supplementary Table S6). It is noted that some genes (focal amplifications of *TRIM46, CCNE1* and *MYC*; homozygous deletions of *CDKN2A, PTEN* and *RB1*) showed a tendency to be more frequently altered in mTNBCs (in particular when compared to the METABRIC cohort alone), although statistical significance was not reached when compared to the two eTNBC cohorts (Fig. 2b).

Using our “discovery strategy”, two new genes *NFIB* (chromosomal region 9p23 which also encompasses *LINC00583*) and *GPC5* (chromosomal region 13q31.3) showed a tendency to be more frequently altered in mTNBCs: *NFIB* was focally amplified in 9.1% of the mTNBCs, as compared to 4.2% in TCGA eTNBCs, and 4.9% in METABRIC eTNBCs. *GPC5* was deleted in 5.3% of the mTNBCs, as compared to 0.6% in TCGA eTNBCs, and in 0.3% of METABRIC eTNBCs (Fig. 2b and Supplementary Table S7).

This low number of more frequently altered in mTNBCs compared to eTNBCs is likely owe to a smaller power of detection (corrected *p* value always not significant), as this was the smallest subsets of TNBCs (in particular the eTNBC TCGA cohort), as compared to ER + /HER2− BCs.

*GPC5* and *NFIB* were not associated with OS both in mTNBCs (Supplementary Table S7) and eTNBCs (data not shown).

### Identification of new mTNBC-specific genes and ER + /HER2− mBC-specific genes, and their prognostic interests in mBC and eBC populations

In this part of our results, we screened our entire genome in order to identify new specific genes of TNBC or ER + /HER2− metastases.

For this purpose, we sought to identify new genes that were differentially altered between the 565 ER + /HER2− mBCs and the 361 mTNBCs, and that were different from the 45 well-known altered region/gene list described in the mBCs (the results of these 45 altered regions/genes in our mBC population are reported in Supplementary Table S8). Six genes (in fold in the Supplementary Table S8) were significantly more amplified in the mTNBCs as compared to the ER + /HER2− BCs, and 9 genes (in bold in the Supplementary Table S8) were significantly more amplified in the ER + /HER2− BCs as compared to the mTNBCs.

By screening the entire genome (“discovery strategy”), 9 new regions/genes were found to be differentially amplified between the 565 ER + /HER2− mBCs and the 361 mTNBCs: 7 regions/genes (*ANGPTL1/ABL2, TFEB/APOBEC2, NFKBIE/VEGFA, RAB23/BMP5, CYP3A4, AKR1B10* and *ASB13*) were more frequently amplified in the mTNBCs and 2 regions/genes (*NFKBIA and TRAF7/NAGPA*) more frequently amplified in the ER + /HER2− (Supplementary Table S9).

The exploration of the prognostic interest of these significant newly discovered genes in the mBC populations showed that none of the 7 mTNBC-specific genes was significantly associated with the prognostic in the mTNBC and the eTNBC populations, as well as none of the 2 ER + /HER2− mBC-specific genes was significantly associated with the prognostic in the ER + /HER2− mBC and in the ER + /HER2− eBC populations (data not shown).

### CNAs analyses in ER + /HER2− mBC population treated with a CDK4/6 inhibitor before biopsies collection

We further assessed whether some CNAs could define a genotype reflecting a mechanism of resistance to CDK4/6i in the ER + /HER2− mBC patients. For this purpose, biopsies collected after CDK4/6i treatment were used. Among these 565 patients, we had the information on the CDK4/6i treatment before biopsies collection for 425 patients: 323 were not treated with CDK4/6i before sample collection, whereas 102 received the CDK4/6i before biopsies collection (Fig. 1). We aimed at identifying genes that were more frequently altered in biopsies collected after CDK4/6i pre-treatment from the 102 ER + /HER2− mBC patients in comparison with the 323 ER + /HER2− mBCs (Fig. 2c).

Among the 45 well-known altered regions/genes, only 2 genes (*ZMIZ1* and *FOXM1*) were found to be more frequently altered (focal amplified) in the cohort of the 102 patients that received the CDK4/6i before samples collection (Table 1 and Supplementary Table S10).

**Table 1:** Known candidate and new discovered genomic alterations in the CDK4 inhibitor-treated population before biopsies collection.

Gene(s)	Chromosomal band	Location	Status^a^	Unpretreated mBC (*n* = 323)	Pretreated mBC (*n* = 102)	Raw *P* value^c^
				%^b^	%^b^	
Candidate						
* ZMIZ1*	10q22.3	80,828,792	Amp	1.5	6.9	**1.31E-02**
* FOXM1*	12p13.33	2,966,847	Amp	1.2	6.9	**5.76E-03**
Discovery						
* AGR2*	7p21.1	16,831,435	Amp	0.9	4.9	**3.11E-02**
* CPNE3 (MMP16)*	8q21.3	87,497,059	Amp	7.1	14.7	**3.22E-02**
* CDK4*	12q14.1	58,141,510	Amp	1.5	9.8	**2.82E-04**
* LGR5*	12q21.1	71,833,550	Amp	3.7	13.7	**5.80E-04**
* NFKBIA*	14q13.2	35,870,717	Amp	3.1	9.8	**1.17E-02**
* CCL1*	17q12	32,687,347	Amp	1.2	6.9	**5.76E-03**
* KCNG1*	20q13.13	49,620,193	Amp	6.5	13.7	**3.51E-02**

^a^Amp: focal amplification.

^b^Percentage of genomic alterations.

^c^Chi-squared test: pretreated versus unpretreated; *P* values in bold are significant.

**Fig. 4:**
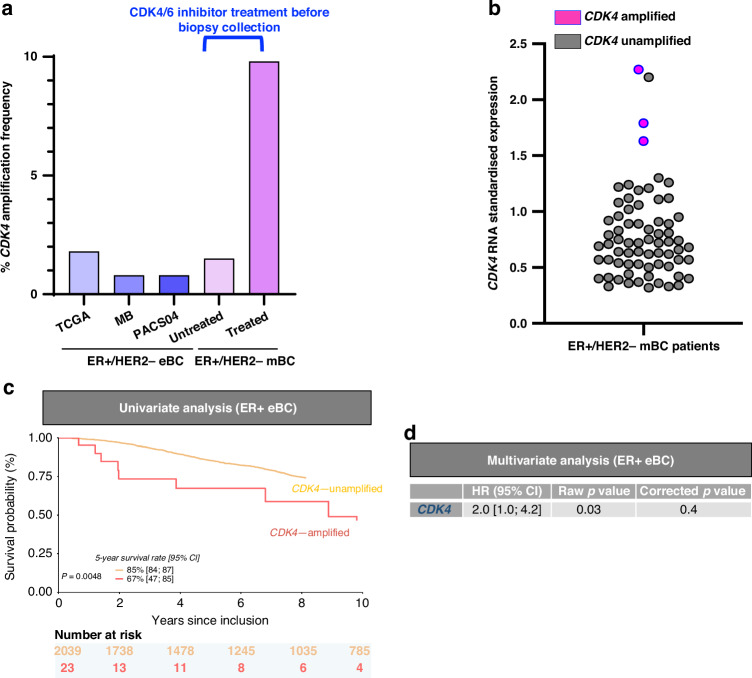
*CDK4* amplification analysis in ER + /HER2- mBC and ER + /HER2− eBC. **a** *CDK4* amplification frequency (%) in ER + /HER2− mBC patients treated or not by CDK4/6i before samples collection and in ER + /HER2− eBC patients. **b** *CDK4* RNA expression by real-time quantitative RT-PCR stratified according the *CDK4* amplification status in ER + /HER2− mBC population. **c** Overall survival in univariable analysis in ER + /HER2− eBC. **d** Overall survival in multivariable analysis in ER + /HER2− eBC.

Using our “discovery strategy”, 7 new genes (*AGR2, CPNE3, CDK4, LGR5, NFKBIA, CCL1* and *KCNG1*) were found to be more frequently altered in the CDK4/6i-treated cohort before samples collection (Table 1). These 7 genes were all focal amplified genes and include the gene *CDK4* itself, the main target of palbociclib. Indeed, *CDK4* was amplified in 9.8% of the ER + /HER2− mBC population treated by CDK4/6i before biopsies collection, as compared to 1.5% in the ER + /HER2− mBC population not treated by CDK4/6i before samples collection (*P* = 2.82E-04) as well as the 3 eBC populations (Table 1 and Fig. 4a).

Among the 425 patients with the information on the CDK4/6i treatment before biopsies collection and the *CDK4* amplification status, 69 have been tested (when RNA was available) for *CDK4* expression by a real-time quantitative RT-PCR: 3 *CDK4*-amplified tumours and 66 *CDK4*-unamplifified tumours. Our results showed that 3 *CDK4*-amplified tumours were among the 4 most *CDK4*-overexpressed tumours (Fig. 4b).

None other known candidate genes for acquired resistance to CDK4/6i was more frequently altered in the CDK4/6i-treated patients before biopsies collection, including *RB1* deletion and *CDK6, CCNE1* and *CCNE2* amplifications (data not shown).

In another step, we explored the prognostic interest of these 9 significant new discovered genes in the ER + /HER2− mBCs and in the ER + /HER2− eBCs.

Interestingly, in ER + /HER2− mBCs, among these 9 amplified genes identified, *FOXM1* was significantly associated (before correction for multiple testing) with poor OS in a multivariable analysis in the CDK4/6i-treated population before samples collection, and not in the not CDK4/6i-treated population (Supplementary Table S11).

It is noted that among these nine amplified genes, only *CDK4* amplification was significantly associated (before correction for multiple testing) with poor OS both in univariable (*P* = 0.0048, Fig. 4c) and multivariable (*P* = 0.03) analysis in the ER + /HER2− eBC patients (Supplementary Table S12). Global results of *CDK4* amplification are reported in Fig. 4.

## Discussion

The aim of our work is the individualisation of biomarkers to guide the therapeutic strategy of breast cancer. First, we highlighted a new prognostic factor in ER + /HER2− mBC: the focal amplification of *TERT* (*Telomerase reverse transcriptase*) that encodes the catalytic subunit of the telomerase. In differentiated cells, the progressive silencing of *TERT* is involved in cell death, whereas in tumour cells, its activation plays a key role in tumour telomeres maintenance and in cell immortality [22]. The increase of the activity level of this enzyme has been described as a pejorative factor in numerous cancer types [23]. Different mechanisms can be involved in *TERT* activation [24]. Among them, three are mostly described: C228T and C250T hotspot mutations of *TERT* promoter, promoter rearrangements, gains of copy numbers and focal amplifications of *TERT*. However, in breast cancer, limited literature is available on *TERT* alterations and mainly at locally advanced settings. The hotspot mutations of *TERT* promoter C228T and C250T appeared to be a rare event in breast cancer with a frequency <0.9% depending on the series and no prognostic correlation has been established so far [25]. In their series of early breast cancers, Gay-Bellile et al. have observed that a gain in copy number of *TERT* was associated with a shorter survival [26]. Our series is the first to describe *TERT* focal amplification in breast metastatic setting and in a specific breast cancer subtype: ER + /HER2−. Even if it is a rare event, observed in 5.8% of the population, this alteration is associated with a shorter OS. Wu et al. have described that *MYC* can directly activate *TERT* by binding to its promoter [27]. Interestingly, we also observed that *MYC* amplification was associated with a pejorative prognosis in both univariable and multivariable analyses. However, the association did not reach significance after multiple testing correction.

CDK4/6i are a cornerstone of the management of ER + /HER2− breast cancers. No biomarker other than HR expression was associated to their development. Nevertheless, 10% of the patients will present an intrinsic resistance, whereas the other will develop inevitably an acquired resistance [28].

One noticeable element of our work is the analysis of the consequence of the exposition to CDK4/6i in ER + /HER2− metastatic breast cancers. We have observed that nine genes were more significantly amplified in biopsies collected after CDK4/6i treatment. Forkhead box M1 (*FOXM1*) is one of them. *FOXM1* encodes an oncogenic transcription factor that plays a crucial role in carcinogenesis, notably in breast cancer [29]. Its upregulation is associated to poor prognosis in breast cancer and also chemoresistance [30]. Hence, FOXM1 inhibitors are considered as interesting compounds to overcome chemoresistance and are currently evaluated in preclinical studies [31].

In our series, we observed more focal amplifications of *FOXM1* in biopsies collected after CDK4/6i treatment population. Interestingly, this amplification was associated to a poor prognosis in this population and had no prognostic value for the CDK4/6i naive population. All these elements support the hypothesis that *FOXM1* may also be involved in CDK4/6i resistance and be a potential target for circumventing it. The first preclinical evaluations are encouraging. Recently, Guillen et al. have observed an enhance effectiveness of FOXM1 inhibitors in combination with CDK4/6i in HR+ cells [32].

*CDK4*, the target of CDK4/6i is also one of the nine genes more amplified after CDK4/6i exposition. It is important to note that this amplification was translated into a RNA expression. *CDK4* amplification has no prognostic value in our series at the metastatic setting but is a poor prognostic factor in early-stage ER + /HER2− patients. Recently, ER + /HER2− early breast cancers with a high risk of recurrence have become eligible to abemaciclib. The “high-risk” definition is only based on clinicohistopathological features, therefore molecular parameters would be valuable.

To date, insight into the molecular pathways involved in the development of resistance to CDK4/6i remains quite limited. Interestingly, none of the commonly putative biomarkers such as *RB1, CCND1, CCNE1* or *CDK6* have shown any prognostic value in the analysed cohorts. These findings suggest that other factors may be involved and highlight the need of further investigations. Furthermore, the discrepancies observed may also be related to methodological differences. The majority of the available data stem from exploratory studies conducted during pivotal phase III trials. It is worth noting that these studies focus on analysing pre-treatment biopsies and establishing correlations with clinical features as post-treatment biopsies were rarely available.

The specificity and the strength of our work lies in the fact that we have a post CDK4/6i treatment biopsy allowing catching biomarkers of acquired resistance.

Our work presents two major limitations. Firstly, it is a retrospective comparative analysis of early and metastatic breast cancers from four different sources. Secondly, we only focus on copy number alterations and did not examine other potential genetic events such as somatic mutations, rearrangement and fusion events. Nevertheless, our findings generate promising hypotheses that warrant further investigations through specific pre-planned well-designed prospective studies.

CDK4/6i have revolutionised the landscape of breast cancer at both metastatic and early stages. A better understanding of the intricate molecular pathways involved in the sensitivity and the resistance to CDK4/6i will pave the way for a more tailored and personalised treatment of breast cancer patients.

## Supplementary information

Supplementary figure 1

Supplementary tables 1-12

## Data Availability

The data that support the findings of this study are available upon request from the authors. The results shown here are in whole or part based upon data generated by the TCGA Research Network: https://www.cancer.gov/tcga.
