# Supplementary material for: Copy number alterations in metastatic and early breast tumours: prognostic and acquired biomarkers of resistance to CDK4/6 inhibitors
Source: Br J Cancer. 2024 Aug 2;131(6):1060–7. doi: 10.1038/s41416-024-02804-6 (PMC11405515; doi:10.1038/s41416-024-02804-6)
Supplement: Supplementary file 1 — Supplementary figure 1 [file 41416_2024_2804_MOESM1_ESM.pdf]

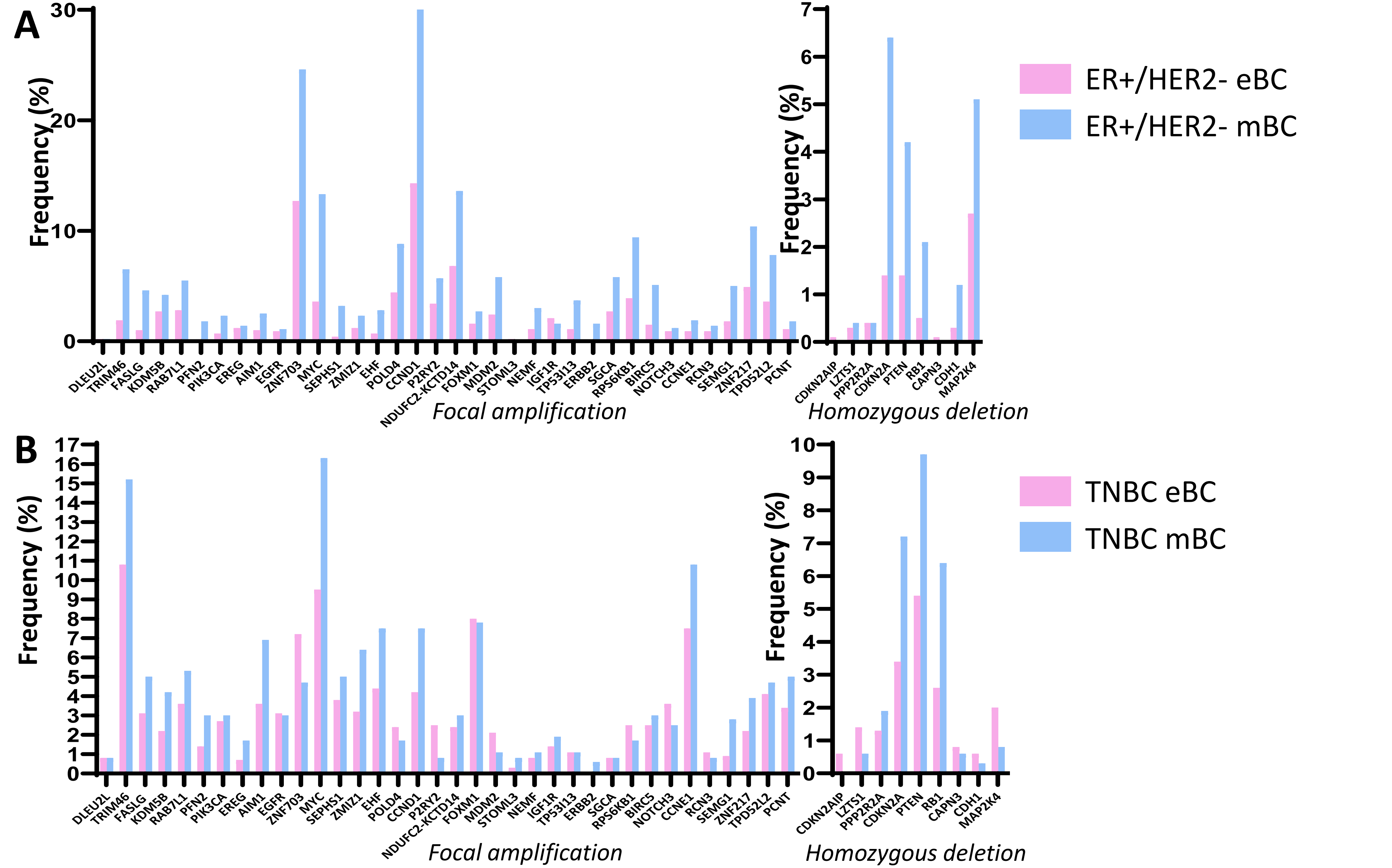

Supplementary Figure 1

Copy Number alterations (CNA) landscapes with focal amplifications and homozygous deletions. **A**, in ER+/HER2- eBC and ER+/HER2- mBC. **B**, in TNBC eBC and TNBC mBC.
