# Supplementary material for: Copy number alterations in metastatic and early breast tumours: prognostic and acquired biomarkers of resistance to CDK4/6 inhibitors
Source: Br J Cancer. 2024 Aug 2;131(6):1060–7. doi: 10.1038/s41416-024-02804-6 (PMC11405515; doi:10.1038/s41416-024-02804-6)
Supplement: Supplementary file 2 — Supplementary tables 1-12 [file 41416_2024_2804_MOESM2_ESM.pdf]

Supplementary Table 1. Patients characteristics in ER+/HER2- and TNBC mBC populations.

Characteristics	TNBC (*n* = 361)	ER+/HER2- (*n* = 565)
	***n* (%)**	***n* (%)**
**Age** median (Q1-Q3)	52 [43-61]	55 [47-64]
**Histological grade**		
SBR1	3 (0.9)	35 (6.6)
SBR2	60 (17.4)	283 (53.2)
SBR3	276 (80)	207 (38.9)
SBR X (non gradable)	6 (1.7)	7 (1.3)
Missing	16	33
**ECOG**†		
ECOG 0	214 (61.5)	259 (50.3)
ECOG 1	132 (37.9)	254 (49.3)
ECOG 2	2 (0.6)	2 (0.4)
Missing	13	50
**Progesteron status**		
Negative	361 (100)	135 (24.6)
Positive	0	413 (75.4)
Missing	0	17
**IHC subtype (primary diagnosis)**		
ER+/HER2-	0	565 (100)
TNBC	361 (100)	0
**Number of metastatic sites (at screening)** median (Q1-Q3)	2 (2-3)	3 (2-4)
**Number of metastatic sites (categorical)**		
1-2	191 (53.7)	248 (45.2)
3-4	133 (37.4)	242 (44.1)
>= 5	32 (9)	59 (10.7)
Missing	5	16
**Presence of liver metastasis**		
No	226 (63.5)	152 (27.7)
Yes	130 (36.5)	397 (72.3)
Missing	5	16
**Presence of lung metastasis**		
No	208 (58.4)	406 (74)
Yes	148 (41.6)	143 (26)
Missing	5	16
**Line of chemotherapy at screening**		
First line	353 (99.2)	522 (95.1)
Second line	3 (0.8)	27 (4.9)
Missing	5	16
**Previous chemotherapy**		
No	104 (29.2)	149 (27.1)
Yes	252 (70.8)	400 (72.9)
Missing	5	16
**Previous adjuvant chemotherapy**		
Anthracyclin + Other	26 (7.3)	56 (10)
Anthracyclin based	69 (19.4)	190 (33.9)
No	228 (64.0)	276 (49.3)
Other chemo/target	33 (9.3)	38 (6.8)
Missing	5	16
**Previous neoadjuvant chemotherapy**		
Anthracyclin + Other	39 (11.0)	55 (10)
Anthracyclin based	103 (28.9)	184 (33.5)
No	207 (58.1)	275 (50.1)
Other chemo/target	7 (2)	35 (6.4)
Missing	5	16
**Previous hormonotherapy**		
No	349 (98)	12 (2.2)
Yes	7 (2)	537 (97.8)
Missing	5	16
**Previous radiotherapy**		
No	118 (33.1)	71 (12.9)
Yes	238 (66.9)	478 (87.1)
Missing	5	16
**Overall survival**		
Alive	111 (31.2)	251 (45.7)
Death	245 (68.8)	298 (54.3)
Missing	5	16
**Follow-up (years)** median (CI* 95%)	2.1 [2.0-2.4]	2.1 [2.1 - 2.3]

† ECOG: Eastern Cooperative Oncology Group Performance score

* CI: confidence interval

Supplementary Table 2. Patient characteristics in ER+/HER2- and TNBC eBC populations.

Characteristics	Metabric		TCGA		PACS04		Total	
	**ER+/HER2- (*n* = 1314)**	**TNBC (*n* = 306)**	**ER+/HER2- (*n* = 605)**	**TNBC (*n* = 165)**	**ER+/HER2- (*n* = 243)**	**NA**	**ER+/HER2- (*n* = 2162)**	**TNBC (*n* = 471)**
	***n* (%)**	***n* (%)**	***n* (%)**	***n* (%)**	*n* (%)		***n* (%)**	***n* (%)**
**Age** (median)	64.0	53.7	59.0	54.0	50.7		61.0	54.0
**Menopause**								
No	196 (15.3)	104 (40.8)	122 (24.6)	34 (26.8)	0 (NaN)		318 (17.9)	138 (36.1)
Yes	1089 (84.7)	151 (59.2)	373 (75.4)	93 (73.2)	0 (NaN)		1462 (82.1)	244 (63.9)
NA	0	0	39	11	243		282	11
Missing	29	51	71	27	0		100	78
**Progesteron status**								
Negative	415 (32.3)	255 (100)	71 (13.3)	138 (100)	43 (18.8)		529 (25.9)	393 (100)
Positive	870 (67.7)	0 (0)	461 (86.7)	0 (0)	186 (81.2)		1517 (74.1)	
Missing	29	51	73	27	14		116	78
**Histological grade**								
1	147 (12)	6 (2.1)	0 (NaN)	0 (NaN)	39 (16.5)		186 (12.7)	6 (2.1)
2	638 (52)	23 (9.1)	0 (NaN)	0 (NaN)	130 (55.1)		768 (52.5)	23 (9.1)
3	442 (36)	230 (90.9)	0 (NaN)	0 (NaN)	67 (28.4)		509 (34.8)	230 (90.9)
Missing	87	53	605	165	7		699	218
**T stage**								
T1 (T ≤ 2 cm)	586 (46)	94 (37.0)	154 (28.8)	37 (26.8)	125 (51.7)		865 (42.2)	131 (33.4)
T2 (2cm < T ≤ 5 cm)	633 (49.7)	143 (56.3)	293 (54.9)	85 (61.6)	105 (43.4)		1031 (50.3)	228 (58.2)
T3 (T > 5 cm)	55 (4.3)	17 (6.7)	87 (16.3)	16 (11.6)	12 (5)		154 (7.5)	33 (8.6)
NA	11	1	0	0	1		12	1
Missing	29	51	71	27	0		100	78
**N stage**								
N0 (0)	714 (55.6)	120 (47.1)	214 (46.4)	85 (66.9)	0 (0)		928 (46.7)	205 (53.7)
N1 (1-3)	391 (30.4)	95 (37.3)	167 (36.2)	25 (19.7)	155 (63.8)		713 (35.8)	120 (31.4)
N2 (4-9)	134 (10.4)	21 (8.2)	49 (10.6)	12 (9.4)	65 (26.7)		248 (12.5)	33 (8.6)
N3 (10+)	46 (3.6)	19 (7.5)	31 (6.7)	5 (3.9)	23 (9.5)		100 (5)	24 (6.3)
Missing	29	51	144	38	0		173	89
**Previous hormonotherapy**								
No	350 (27.2)	195 (76.5)	0 (NaN)	0 (NaN)	2 (0.8)		352 (23.1	195 (76.5)
Yes	935 (72.8)	60 (23.5)	0 (NaN)	0 (NaN)	237 (99.2)		1172 (76.9)	60 (23.5)
Missing	29	51	605	165	4		638	216
**Previous radiotherapy**								
No	546 (42.5)	72 (28.2)	194 (39.6)	53 (42.4)	7 (2.9)		747 (37)	125 (32.9)
Yes	739 (57.5)	183 (71.8)	296 (60.4)	72 (57.6)	236 (97.1)		1271 (63)	255 (67.1)
Missing	29	51	115	40	0		144	91
**Overall survival**								
0	537 (41.8)	117 (45.9)	484 (90.6)	117 (84.8)	204 (84)		1225 (59.4)	234 (59.5)
1	748 (58.2)	138 (54.1)	50 (9.4)	21 (15.2)	39 (16)		837 (40.6)	159 (40.5)
Missing	29	51	71	27	0		100	78

Supplementary Table 3. Genomic alterations in the ER+/HER2- populations.

Gene(s)	Chromosomal band	Location	Status[a]	mBC (n = 565)	TCGA (n = 605)			Metabric (n = 1314)			PACS04 (n = 243)		
				%[b]	%[b]	Raw p-value[c]	Corrected p-value[d]	%[b]	Raw p-value[c]	Corrected p-value[d]	%[b]	Raw p-value[c]	Corrected p-value[d]
**Know candidate genes (n = 45)**													
DLEU2L	1p31.3	64014588	amp	0,2	0,3	1,00E+00	1,00E+00	0,2	1,00E+00	1,00E+00	0,0	1,00E+00	1,00E+00
TRIM46	1q22	155145873	amp	6,5	1,8	**8,53E-05**	**4,68E-03**	2,1	**3,05E-06**	**5,98E-05**	1,6	**6,20E-03**	1,17E-01
FASLG (TNFSF18/TNFSF4)	1q24.3	172628154	amp	4,6	1,5	**3,15E-03**	5,49E-02	1,1	**5,74E-06**	**1,01E-04**	0,4	**4,72E-03**	1,12E-01
KDM5B	1q32.1	202696526	amp	4,2	3,0	3,12E-01	8,49E-01	2,3	**2,87E-02**	1,09E-01	2,9	**4,67E-01**	9,54E-01
RAB7L1	1q32.1	205737114	amp	5,5	2,3	**7,63E-03**	1,05E-01	2,5	**1,80E-03**	**1,25E-02**	3,7	**3,71E-01**	9,02E-01
PFN2	3q25.1	149682691	amp	1,8	0,0	**2,99E-03**	5,39E-02	0,4	**4,79E-03**	**2,72E-02**	0,0	**8,19E-02**	4,46E-01
PIK3CA	3q26.32	178865902	amp	2,3	1,2	2,00E-01	6,92E-01	0,2	**2,56E-05**	**3,71E-04**	0,8	**2,53E-01**	7,22E-01
EREG	4q13.3	75230860	amp	1,4	1,3	1,00E+00	1,00E+00	0,9	4,66E-01	8,05E-01	1,2	1,00E+00	1,00E+00
AIM1	6q21	106959730	amp	2,5	1,7	4,30E-01	9,56E-01	0,6	**1,28E-03**	**9,62E-03**	0,8	**2,03E-01**	6,75E-01
EGFR	7p11.2	55086714	amp	1,1	0,7	6,70E-01	1,00E+00	0,5	3,34E-01	6,53E-01	1,6	7,33E-01	1,00E+00
ZNF703 (FGFR1)	8p11.23	37553269	amp	24,6	15,7	**1,92E-04**	**7,97E-03**	10,8	**2,57E-14**	**5,98E-12**	11,5	**3,86E-05**	**1,23E-02**
MYC	8q24.21	128747680	amp	13,3	4,3	**8,35E-08**	**3,47E-05**	3,1	**1,21E-16**	**6,08E-14**	3,3	**3,19E-05**	**1,23E-02**
SEPHS1	10p13	13359424	amp	3,2	0,7	**3,06E-03**	5,41E-02	0,6	**3,05E-05**	**4,30E-04**	0,0	**1,06E-02**	1,43E-01
ZMIZ1	10q22.3	80828792	amp	2,3	1,7	5,57E-01	1,00E+00	1,2	1,23E-01	3,30E-01	0,8	2,53E-01	7,22E-01
EHF	11p13	34642640	amp	2,8	0,8	**1,82E-02**	1,79E-01	0,8	**9,38E-04**	**7,42E-03**	0,4	**5,35E-02**	3,59E-01
POLD4	11q13.2	67118248	amp	8,8	5,5	**3,18E-02**	2,49E-01	4,7	**7,73E-04**	**6,27E-03**	2,9	**3,87E-03**	1,02E-01
CCND1	11q13.3	69455855	amp	30,1	15,7	**6,44E-09**	**5,24E-06**	14,0	**4,96E-16**	**1,80E-13**	13,2	**5,59E-07**	**1,43E-03**
P2RY2	11q13.4	72929343	amp	5,7	3,8	1,72E-01	6,57E-01	3,4	**3,42E-02**	1,27E-01	2,9	**1,30E-01**	5,56E-01
NDUFC2-KCTD14	11q14.1	77728017	amp	13,6	6,0	**1,40E-05**	**1,50E-03**	6,7	**1,76E-06**	**3,88E-05**	7,8	**2,63E-02**	2,41E-01
FOXM1	12p13.33	2966847	amp	2,7	2,0	5,69E-01	1,00E+00	1,9	3,89E-01	7,40E-01	0,8	1,63E-01	6,16E-01
MDM2	12q15	69201956	amp	5,8	3,8	1,35E-01	5,72E-01	2,2	**9,51E-05**	**1,13E-03**	1,2	**6,45E-03**	1,17E-01
STOML3	13q13.3	39540062	amp	0,0	0,3	5,09E-01	9,95E-01	0,2	8,76E-01	1,00E+00	0,0	NA	NA
NEMF	14q21.3	50249997	amp	3,0	1,2	**4,27E-02**	2,92E-01	1,4	**3,73E-02**	1,34E-01	0,8	**1,04E-01**	5,01E-01
IGF1R	15q26.3	99192200	amp	1,6	2,1	6,28E-01	1,00E+00	1,8	9,62E-01	1,00E+00	2,5	5,74E-01	1,00E+00
TP53I13	17q11.2	27893070	amp	3,7	2,3	2,17E-01	7,04E-01	0,5	**5,27E-07**	**1,44E-05**	0,4	**1,59E-02**	1,80E-01
ERBB2	17q12	37844167	amp	1,6	0,0	**5,41E-03**	8,21E-02	0,0	**2,42E-05**	**3,53E-04**	0,0	**1,07E-01**	5,01E-01
SGCA	17q21.33	48241575	amp	5,8	3,8	1,35E-01	5,72E-01	2,5	**5,44E-04**	**4,96E-03**	1,6	**1,50E-02**	1,74E-01
RPS6KB1	17q23.1	57970447	amp	9,4	6,0	**3,56E-02**	2,54E-01	3,4	**1,87E-07**	**6,23E-06**	2,5	**9,15E-04**	5,70E-02
BIRC5	17q25.3	76210267	amp	5,1	2,0	**5,64E-03**	8,50E-02	1,6	**2,56E-05**	**3,71E-04**	0,8	**6,43E-03**	1,17E-01
NOTCH3	19p13.12	15270444	amp	1,2	1,3	1,00E+00	1,00E+00	1,1	9,29E-01	1,00E+00	0,4	4,83E-01	9,60E-01
CCNE1	19q12	30302805	amp	1,9	1,5	7,04E-01	1,00E+00	0,8	6,92E-02	2,09E-01	0,4	1,81E-01	6,16E-01
RCN3	19q13.33	50030875	amp	1,4	0,8	4,95E-01	9,95E-01	1,4	1,00E+00	1,00E+00	0,4	3,78E-01	9,02E-01
SEMG1	20q13.12	43835638	amp	5,0	2,5	**3,62E-02**	2,55E-01	1,3	**4,30E-06**	**7,93E-05**	1,6	**4,39E-02**	3,25E-01
ZNF217	20q13.2	52183604	amp	10,4	6,6	**2,46E-02**	2,18E-01	4,3	**5,16E-07**	**1,42E-05**	3,7	**2,48E-03**	8,59E-02
TPD52L2	20q13.33	62496596	amp	7,8	5,0	6,20E-02	3,53E-01	3,0	**5,63E-06**	**1,00E-04**	2,9	**1,34E-02**	1,62E-01
PCNT	21q22.3	47744036	amp	1,8	1,5	8,81E-01	1,00E+00	0,9	1,77E-01	4,17E-01	0,8	4,82E-01	9,60E-01
CDKN2AIP	4q35.1	184365744	del	0,0	0,2	1,00E+00	1,00E+00	0,1	1,00E+00	1,00E+00	0,0	NA	NA
LZTS1	8p21.3	20103676	del	0,4	0,7	7,45E-01	1,00E+00	0,2	1,00E+00	1,00E+00	0,0	8,75E-01	1,00E+00
PPP2R2A	8p21.2	26149007	del	0,4	0,5	1,00E+00	1,00E+00	0,3	1,00E+00	1,00E+00	0,4	1,00E+00	1,00E+00
CDKN2A	9p21.3	21967751	del	6,4	2,0	**2,79E-04**	**8,47E-03**	0,8	**5,84E-12**	**2,73E-10**	1,2	**3,23E-03**	1,10E-01
PTEN	10q23.31	89622870	del	4,2	1,5	**7,52E-03**	1,44E-01	1,4	**2,16E-04**	**3,55E-03**	1,2	**4,86E-02**	7,97E-01
RB1	13q14.2	48877887	del	2,1	0,7	5,73E-02	7,65E-01	0,3	**2,50E-04**	**4,08E-03**	0,4	**1,42E-01**	1,00E+00
CAPN3	15q15.1	42640301	del	0,0	0,2	1,00E+00	1,00E+00	0,0	NA	NA	0,0	NA	NA
CDH1	16q22.1	68771128	del	1,2	0,2	6,12E-02	7,65E-01	0,2	**1,57E-02**	1,55E-01	0,4	**4,83E-01**	1,00E+00
MAP2K4	17p12	11924141	del	5,1	2,6	**3,95E-02**	5,57E-01	1,8	**7,97E-05**	**1,41E-03**	3,7	**4,85E-01**	1,00E+00
**New discovered genes**													
GJC2	1q42.13	228337553	Amp	5,0	0,5	**5,01E-06**	**7,29E-04**	1,0	**1,75E-07**	**5,89E-06**	0,4	**2,90E-03**	9,26E-02
TERT	5p15.33	1253262	Amp	5,8	1,8	**5,40E-04**	**1,55E-02**	1,4	**2,32E-07**	**7,31E-06**	0,0	**2,59E-04**	**3,47E-02**
RAD21	8q24.11	117858174	Amp	14,0	4,5	**2,59E-08**	**1,58E-05**	2,2	**2,57E-23**	**1,26E-19**	5,3	**6,22E-04**	**4,79E-02**
ST3GAL1 (NDRG1/WISP1)	8q24.22	134467091	Amp	9,4	2,5	**8,80E-07**	**1,85E-04**	2,0	**5,77E-13**	**1,14E-10**	2,5	**9,15E-04**	5,70E-02
SOCS1	16p13.13	11348262	Amp	4,1	1,0	**1,39E-03**	**3,07E-02**	1,0	**1,83E-05**	**2,76E-04**	0,0	**3,07E-03**	9,39E-02

[a] : Amp : Focal amplification; Del : Homozygous deletion

[b] : Percentage of genomic alterations

[c] : Chi-squared test : TCGA (or Metabric or PACS04) *versus* mBC ; genes and p-values in bold are significant

[d] : Corrected Chi-squared test : TCGA (or Metabric or PACS04) *versus* mBC ; genes and p-values in bold are significant

NA: Not Applicable. Frequencies at 0% in both populations (mBC and eBC)

Supplementary Table 4. Prognostic effect of copy number alterations in the ER+/HER2- mBC population.

				Univariate (OS)	*Multivariate (OS)*		
**Gene(s)**	**Chromosomal band**	**Location**	**Status[a]**	**p-value[b]**	**HR (95% CI)**	**Raw p-value[b]**	**Corrected p-value[b]**
TRIM46	1q22	155145873	Amp	0,38	1.0 [0.5 ; 1.7]	0,16	1.00
FASLG (TNFSF18/TNFSF4)	1q24.3	172628154	Amp	0,49	0.7 [0.3 ; 1.4]	0,3	1.00
ZNF703 (FGFR1)	8p11.23	37553269	Amp	0,09	1.3 [1.0 ; 1.7]	0,09	1.00
MYC	8q24.21	128747680	Amp	**0,02**	1.5 [1.0 ; 2.3]	**0,03**	0,53
SEPHS1	10p13	13359424	Amp	0,55	0.8 [0.4 ; 1.6]	0,46	1.00
POLD4	11q13.2	67118248	Amp	0,96	1.1 [0.7 ; 1.7]	0,98	1.00
CCND1	11q13.3	69455855	Amp	0,57	1.1 [0.8 ; 1.4]	0,75	1.00
NDUFC2-KCTD14	11q14.1	77728017	Amp	0,85	0.8 [0.4 ; 1.3]	0,84	1.00
RPS6KB1	17q23.1	57970447	Amp	0,38	0.8 [0.5 ; 1.2]	0,25	1.00
BIRC5	17q25.3	76210267	Amp	0,86	1.2 [0.6 ; 2.1]	0,72	1.00
SEMG1	20q13.12	43835638	Amp	0,64	1.0 [0.5 ; 1.9]	0,92	1.00
ZNF217	20q13.2	52183604	Amp	0,84	1.2 [0.8 ; 1.8]	0,79	1.00
CDKN2A	9p21.3	21967751	Del	0,22	1.2 [0.8 ; 1.9]	0,17	1.00
PTEN	10q23.31	89622870	Del	0,22	1.5 [0.9 ; 2.5]	0,13	1.00
GJC2	1q42.13	228337553	Amp	0,34	0.8 [0.4 ; 1.5]	0,18	1.00
TERT	5p15.33	1253262	Amp	**<0.0001**	2.6 [1.6 ; 4.0]	**<0.001**	**0.01**
RAD21	8q24.11	117858174	Amp	**0,03**	1.4 [0.9 ; 2.0]	0,07	1.00
ST3GAL1 (NDRG1/WISP1)	8q24.22	134467091	Amp	0,67	0.6 [0.4 ; 1.0]	0,52	1.00
SOCS1	16p13.13	11348262	Amp	0,58	0.8 [0.4 ; 1.6]	0,26	1.00

[a] : Amp : Focal amplification; Del : Homozygous deletion

[b] : p-values in bold are significant

Supplementary Table 5. Genomic regions with prognostic effect of copy number alterations in the ER+/HER2- eBC population.

				Univariate (OS)	***Multivariate (OS)***		
**Gene(s)**	**Chromosomal band**	**Location**	**Status[a]**	**p-value[b]**	**HR (95% CI)**	**Raw p-value[b]**	**Corrected p-value**
TRIM46	1q22	155145873	Amp	0,38	1.1 [0.7 ; 1.8]	0,38	1,00
FASLG (TNFSF18/TNFSF4)	1q24.3	172628154	Amp	0,47	1.2 [0.6 ; 2.2]	0,39	1,00
ZNF703 (FGFR1)	8p11.23	37553269	Amp	0,20	1.1 [0.9 ; 1.4]	0,35	1,00
MYC	8q24.21	128747680	Amp	0,28	1.2 [0.8 ; 1.9]	0,61	1,00
SEPHS1	10p13	13359424	Amp	0,65	1.0 [0.4 ; 2.5]	0,21	1,00
POLD4	11q13.2	67118248	Amp	0,74	0.9 [0.6 ; 1.3]	0,49	1,00
CCND1	11q13.3	69455855	Amp	0,37	1.2 [0.9 ; 1.5]	0,58	1,00
NDUFC2-KCTD14	11q14.1	77728017	Amp	0,81	0.8 [0.5 ; 1.2]	0,88	1,00
RPS6KB1	17q23.1	57970447	Amp	0,45	0.7 [0.4 ; 1.0]	**0,05**	1,00
BIRC5	17q25.3	76210267	Amp	0,33	1.3 [0.7 ; 2.2]	0,65	1,00
SEMG1	20q13.12	43835638	Amp	0,51	0.8 [0.4 ; 1.8]	**<0.01**	0,20
ZNF217	20q13.2	52183604	Amp	0,49	1.0 [0.7 ; 1.6]	0,11	1,00
CDKN2A	9p21.3	21967751	Del	**<0.01**	2.0 [1.1 ; 3.6]	**0,02**	0,42
PTEN	10q23.31	89622870	Del	**0,02**	1.5 [0.9 ; 2.6]	**0,03**	0,63
GJC2	1q42.13	228337553	Amp	**<0.01**	2.7 [1.4 ; 5.2]	**0,02**	0,42
TERT	5p15.33	1253262	Amp	0,13	1.5 [0.8 ; 2.6]	0,46	1,00
RAD21	8q24.11	117858174	Amp	0,70	1.0 [0.6 ; 1.6]	0,14	1,00
ST3GAL1 (NDRG1/WISP1)	8q24.22	134467091	Amp	0,27	NA	0,09	1,00
SOCS1	16p13.13	11348262	Amp	0,29	0.6 [0.3 ; 1.5]	**0,03**	0,53

[a] : Amp : Focal amplification; Del : Homozygous deletion

[b] : p-values in bold are significant

NA: Not Applicable.

Supplementary Table 6. Known candidate genomic alterations in the TNBC populations.

Gene(s)	Chromosomal band	Location	Status[a]	mBC (*n* = 361)	TCGA (*n* = 165)		Metabric (*n* = 306)	
				%[b]	%[b]	Raw p-value[c]	%[b]	Raw p-value[c]
DLEU2L	1p31.3	64014588	amp	0,8	0,0	5,82E-01	1,6	5,54E-01
TRIM46	1q22	155145873	amp	15,2	13,3	6,60E-01	8,2	**7,38E-03**
FASLG (TNFSF18/TNFSF4)	1q24.3	172628154	amp	5,0	3,0	4,31E-01	3,3	3,63E-01
KDM5B	1q32.1	202696526	amp	4,2	2,4	4,62E-01	2,0	1,63E-01
RAB7L1	1q32.1	205737114	amp	5,3	3,6	5,53E-01	3,6	3,96E-01
PFN2	3q25.1	149682691	amp	3,0	0,6	1,54E-01	2,3	7,16E-01
PIK3CA	3q26.32	178865902	amp	3,0	2,4	9,08E-01	2,9	1,00E+00
EREG	4q13.3	75230860	amp	1,7	0,0	2,21E-01	1,3	9,55E-01
AIM1	6q21	106959730	amp	6,9	3,6	1,98E-01	3,6	8,46E-02
EGFR	7p11.2	55086714	amp	3,0	3,0	1,00E+00	3,3	1,00E+00
ZNF703 (FGFR1)	8p11.23	37553269	amp	4,7	9,1	7,94E-02	5,2	8,97E-01
MYC	8q24.21	128747680	amp	16,3	12,1	2,60E-01	6,9	**2,77E-04**
SEPHS1	10p13	13359424	amp	5,0	2,4	2,60E-01	5,2	1,00E+00
ZMIZ1	10q22.3	80828792	amp	6,4	2,4	9,09E-02	3,9	2,15E-01
EHF	11p13	34642640	amp	7,5	3,6	1,36E-01	5,2	3,07E-01
POLD4	11q13.2	67118248	amp	1,7	2,4	8,03E-01	2,3	7,63E-01
CCND1	11q13.3	69455855	amp	7,5	4,2	2,26E-01	4,2	1,12E-01
P2RY2	11q13.4	72929343	amp	0,8	3,0	1,26E-01	2,0	3,56E-01
NDUFC2-KCTD14	11q14.1	77728017	amp	3,0	1,8	6,03E-01	2,9	1,00E+00
FOXM1	12p13.33	2966847	amp	7,8	7,3	9,87E-01	8,8	7,20E-01
MDM2	12q15	69201956	amp	1,1	1,8	8,03E-01	2,3	3,75E-01
STOML3	13q13.3	39540062	amp	0,8	0,0	5,82E-01	0,7	1,00E+00
NEMF	14q21.3	50249997	amp	1,1	0,6	9,47E-01	1,0	1,00E+00
IGF1R	15q26.3	99192200	amp	1,9	1,2	8,15E-01	1,6	9,98E-01
TP53I13	17q11.2	27893070	amp	1,1	0,6	9,47E-01	1,6	8,03E-01
ERBB2	17q12	37844167	amp	0,6	0,0	8,46E-01	0,0	5,53E-01
SGCA	17q21.33	48241575	amp	0,8	0,6	1,00E+00	1,0	1,00E+00
RPS6KB1	17q23.1	57970447	amp	1,7	3,0	4,91E-01	2,0	1,00E+00
BIRC5	17q25.3	76210267	amp	3,0	3,0	1,00E+00	2,0	5,22E-01
NOTCH3	19p13.12	15270444	amp	2,5	3,6	6,54E-01	3,6	5,46E-01
CCNE1	19q12	30302805	amp	10,8	9,1	6,56E-01	5,9	**3,35E-02**
RCN3	19q13.33	50030875	amp	0,8	1,2	1,00E+00	1,0	1,00E+00
SEMG1	20q13.12	43835638	amp	2,8	1,2	4,26E-01	0,7	7,89E-02
ZNF217	20q13.2	52183604	amp	3,9	1,8	3,30E-01	2,6	4,88E-01
TPD52L2	20q13.33	62496596	amp	4,7	3,6	7,43E-01	4,6	1,00E+00
PCNT	21q22.3	47744036	amp	5,0	4,2	8,80E-01	2,6	1,69E-01
CDKN2AIP	4q35.1	184365744	del	0,0	1,2	1,83E-01	0,0	NA
LZTS1	8p21.3	20103676	del	0,6	1,8	3,67E-01	1,0	8,53E-01
PPP2R2A	8p21.2	26149007	del	1,9	1,2	8,15E-01	1,3	7,39E-01
CDKN2A	9p21.3	21967751	del	7,2	4,2	2,69E-01	2,6	**1,22E-02**
PTEN	10q23.31	89622870	del	9,7	7,9	6,11E-01	2,9	**8,22E-04**
RB1	13q14.2	48877887	del	6,4	3,6	2,85E-01	1,6	**4,42E-03**
CAPN3	15q15.1	42640301	del	0,6	1,2	7,91E-01	0,3	1,00E+00
CDH1	16q22.1	68771128	del	0,3	0,6	1,00E+00	0,7	8,86E-01
MAP2K4	17p12	11924141	del	0,8	2,4	2,85E-01	1,6	5,54E-01

[a] : Amp : Focal amplification; Del : Homozygous deletion

[b] : Percentage of genomic alterations

[c] : Chi-squared test : TCGA (or Metabric) *versus* mBC ; genes and p-values in bold are significant

NA: Not Applicable. Frequencies at 0% in both populations (mBC and Metabric)

Supplementary Table 7. Genomic regions with prognostic effect of copy number alterations in the TNBC mBC population.

Gene(s)	Chromosomal band	Location	Status[a]	mBC (*n* = 361)	TCGA (*n* = 165)		Metabric (*n* = 306)		*Univariate (OS)*	*Multivariate (OS)*		
				%[b]	%[b]	Raw p-value[c]	%[b]	Raw p-value[c]	P-value	HR (95% CI)	Raw p-value	Corrected p-value
NFIB (LINC00583)	9p23	14081842	Amp	9,1	4,2	**7,35E-02**	5	**4,99E-02**	0,93	0.9 [0.4 ; 1.9]	6,20E-01	1,00
GPC5	13q31.3	92050929	Del	5,3	0,6	**1,90E-02**	0,3	**5,00E-04**	0,45	1.9 [0.4 ; 7.9]	4,10E-01	0,82

[a] : Amp : Focal amplification; Del : Homozygous deletion

[b] : Percentage of genomic alterations

[c] : Chi-squared test: TCGA (or Metabric) *versus* mBC; p-values in bold are significant

Supplementary Table 8. Known candidate genomic alterations in ER+/HER2- mBC in comparison with mTNBC.

Gene(s)	Chromosomal band	Location	Status[a]	ER+/HER2- mBC (*n* = 565) %[b]	mTNBC (*n* = 361) %[b]	Raw p-value[c]	Corrected p-value
DLEU2L	1p31.3	64014588	amp	0,2	0,8	3,34E-01	7,64E-01
TRIM46	1q22	155145873	amp	6,5	**15,2**	**2,70E-05**	**1,28E-03**
FASLG (TNFSF18/TNFSF4)	1q24.3	172628154	amp	4,6	5,0	9,13E-01	1,00E+00
KDM5B	1q32.1	202696526	amp	4,2	4,2	1,00E+00	1,00E+00
RAB7L1	1q32.1	205737114	amp	5,5	5,3	1,00E+00	1,00E+00
PFN2	3q25.1	149682691	amp	1,8	3,0	2,95E-01	7,58E-01
PIK3CA	3q26.32	178865902	amp	2,3	3,0	6,28E-01	1,00E+00
EREG	4q13.3	75230860	amp	1,4	1,7	9,81E-01	1,00E+00
AIM1	6q21	106959730	amp	2,5	**6,9**	**1,82E-03**	**2,78E-02**
EGFR	7p11.2	55086714	amp	1,1	3,0	5,19E-02	2,69E-01
ZNF703 (FGFR1)	8p11.23	37553269	amp	**24,6**	4,7	**6,29E-15**	**2,06E-11**
MYC	8q24.21	128747680	amp	13,3	16,3	2,31E-01	6,86E-01
SEPHS1	10p13	13359424	amp	3,2	5,0	2,27E-01	6,76E-01
ZMIZ1	10q22.3	80828792	amp	2,3	**6,4**	**3,17E-03**	**4,24E-02**
EHF	11p13	34642640	amp	2,8	**7,5**	**1,82E-03**	**2,78E-02**
POLD4	11q13.2	67118248	amp	**8,8**	1,7	**1,47E-05**	**8,55E-04**
CCND1	11q13.3	69455855	amp	**30,1**	7,5	**4,78E-16**	**4,70E-12**
P2RY2	11q13.4	72929343	amp	**5,7**	0,8	**3,38E-04**	**8,68E-03**
NDUFC2-KCTD14	11q14.1	77728017	amp	**13,6**	3,0	**1,61E-07**	**2,98E-05**
FOXM1	12p13.33	2966847	amp	2,7	**7,8**	**5,86E-04**	**1,31E-02**
MDM2	12q15	69201956	amp	**5,8**	1,1	**6,40E-04**	**1,40E-02**
STOML3	13q13.3	39540062	amp	0,0	0,8	1,15E-01	4,44E-01
NEMF	14q21.3	50249997	amp	3,0	1,1	9,52E-02	3,91E-01
IGF1R	15q26.3	99192200	amp	1,6	1,9	8,92E-01	1,00E+00
TP53I13	17q11.2	27893070	amp	3,7	1,1	**2,92E-02**	1,89E-01
ERBB2	17q12	37844167	amp	1,6	0,6	2,66E-01	7,58E-01
SGCA	17q21.33	48241575	amp	**5,8**	0,8	**2,41E-04**	**6,71E-03**
RPS6KB1	17q23.1	57970447	amp	**9,4**	1,7	**5,31E-06**	**3,89E-04**
BIRC5	17q25.3	76210267	amp	5,1	3,0	1,75E-01	5,54E-01
NOTCH3	19p13.12	15270444	amp	1,2	2,5	2,42E-01	7,06E-01
CCNE1	19q12	30302805	amp	1,9	**10,8**	**1,46E-08**	**5,02E-06**
RCN3	19q13.33	50030875	amp	1,4	0,8	6,24E-01	1,00E+00
SEMG1	20q13.12	43835638	amp	5,0	2,8	1,43E-01	5,28E-01
ZNF217	20q13.2	52183604	amp	**10,4**	3,9	**4,83E-04**	**1,15E-02**
TPD52L2	20q13.33	62496596	amp	7,8	4,7	8,80E-02	3,83E-01
PCNT	21q22.3	47744036	amp	1,8	5,0	**9,58E-03**	8,77E-02
CDKN2AIP	4q35.1	184365744	del	0,0	0,0	2,76E-01	7,58E-01
LZTS1	8p21.3	20103676	del	0,4	0,6	1,00E+00	1,00E+00
PPP2R2A	8p21.2	26149007	del	0,4	1,9	8,21E-01	1,00E+00
CDKN2A	9p21.3	21967751	del	6,4	7,2	**5,37E-03**	6,11E-02
PTEN	10q23.31	89622870	del	4,2	9,7	2,96E-01	7,58E-01
RB1	13q14.2	48877887	del	2,1	6,4	8,21E-01	1,00E+00
CAPN3	15q15.1	42640301	del	0,0	0,6	9,51E-01	1,00E+00
CDH1	16q22.1	68771128	del	1,2	0,3	6,95E-01	1,00E+00
MAP2K4	17p12	11924141	del	5,1	0,8	1,00E+00	1,00E+00

[a] : Amp : Focal amplification; Del : Homozygous deletion

[b] : Percentage of genomic alterations; values in bold correspond to genes statistically more amplified with corrected pvalue

[c] : Chi-squared test : mTNBC *versus ER+/HER2-* mBC; p-values in bold are significant

Supplementary Table 9. New specific genomic alterations in the ER-/HER2- mBC or mTNBC population

Gene(s)	Chromosomal band	Location	Status[a]	ER+/HER2- mBC (*n* = 565) %[b]	mTNBC (*n* = 361) %[b]	Raw p-value[c]
**New specific genomic alterations in the mTNBC**						
ANGPTL1 (ABL2)	1q25.2	178818840	Amp	3,2	8,8	**3,49E-04**
TFEB (APOBEC2)	6p21.1	41651716	Amp	0,7	4,7	**1,68E-04**
NFKBIE (VEGFA)	6p21.1	44225903	Amp	0,5	4,4	**1,20E-04**
RAB23 (BMP5)	6p11.2	57053607	Amp	0,9	4,2	**1,89E-03**
CYP3A4	7q22.1	99354604	Amp	0,4	3,6	**3,84E-04**
AKR1B10	7q33	134212344	Amp	0,4	3,9	**1,73E-04**
ASB13	10p15.1	5680830	Amp	3,2	8,9	**3,44E-04**
**New specific genomic alterations in the ER+/HER2- mBC**						
NFKBIA	14q13.2	35870717	Amp	4,2	0,3	**6,08E-04**
TRAF7 (NAGPA)	16p13.3	2205699	Amp	5,3	0,6	**2,33E-04**

[a] : Amp : Focal amplification

[b] : Percentage of genomic alterations

[c] : Chi-squared test : mTNBC *versus* ER+/HER2- mBC ; p-values in bold are significant

Supplementary Table 10. Known candidates genomic alterations in the ER+/HER2- populationby CDK4 inhibitor before biopsies collection.

Gene(s)	Chromosomal band	Location	Status[a]	non pre-treated mBC (*n* = 323) %[b]	pre-treated mBC (*n* = 102) %[b]	Raw p-value[c]
DLEU2L	1p31.3	64014588	amp	0.3	0.0	1.00E+00
TRIM46	1q22	155145873	amp	8.7	4.9	3.04E-01
FASLG (TNFSF18/TNFSF4)	1q24.3	172628154	amp	5.9	5.9	1.00E+00
KDM5B	1q32.1	202696526	amp	5.3	3.9	7.77E-01
RAB7L1	1q32.1	205737114	amp	5.9	8.8	4.15E-01
PFN2	3q25.1	149682691	amp	2.5	1.0	6.03E-01
PIK3CA	3q26.32	178865902	amp	2.2	2.9	9.40E-01
EREG	4q13.3	75230860	amp	1.5	2.0	1.00E+00
AIM1	6q21	106959730	amp	1.9	4.9	1.83E-01
EGFR	7p11.2	55086714	amp	1.2	1.0	1.00E+00
ZNF703 (FGFR1)	8p11.23	37553269	amp	25.1	31.4	2.60E-01
MYC	8q24.21	128747680	amp	13.0	16.7	4.42E-01
SEPHS1	10p13	13359424	amp	3.7	3.9	1.00E+00
ZMIZ1	10q22.3	80828792	amp	1.5	6.9	**1.31E-02**
EHF	11p13	34642640	amp	2.8	3.9	8.02E-01
POLD4	11q13.2	67118248	amp	10.5	8.8	7.57E-01
CCND1	11q13.3	69455855	amp	30.0	29.4	1.00E+00
P2RY2	11q13.4	72929343	amp	4.6	8.8	1.78E-01
NDUFC2-KCTD14	11q14.1	77728017	amp	13.6	12.7	9.52E-01
FOXM1	12p13.33	2966847	amp	1.2	6.9	**5.76E-03**
MDM2	12q15	69201956	amp	5.3	10.8	8.35E-02
STOML3	13q13.3	39540062	amp	0.0	0.0	NA
NEMF	14q21.3	50249997	amp	4.0	2.0	4.98E-01
IGF1R	15q26.3	99192200	amp	1.2	2.0	9.54E-01
TP53I13	17q11.2	27893070	amp	3.1	3.9	9.29E-01
ERBB2	17q12	37844167	amp	2.2	2.0	1.00E+00
SGCA	17q21.33	48241575	amp	6.2	6.9	9.93E-01
RPS6KB1	17q23.1	57970447	amp	9.3	10.8	8.00E-01
BIRC5	17q25.3	76210267	amp	4.6	7.8	3.20E-01
NOTCH3	19p13.12	15270444	amp	1.9	0.0	3.65E-01
CCNE1	19q12	30302805	amp	2.2	1.0	7.26E-01
RCN3	19q13.33	50030875	amp	1.9	0.0	3.65E-01
SEMG1	20q13.12	43835638	amp	5.0	8.8	2.28E-01
ZNF217	20q13.2	52183604	amp	10.8	11.8	9.36E-01
TPD52L2	20q13.33	62496596	amp	8.7	9.8	8.80E-01
PCNT	21q22.3	47744036	amp	1.9	2.9	7.89E-01
CDKN2AIP	4q35.1	184365744	del	0.0	0.0	NA
LZTS1	8p21.3	20103676	del	n	0.0	NA
PPP2R2A	8p21.2	26149007	del	0.0	0.0	NA
CDKN2A	9p21.3	21967751	del	6.5	2.9	2.66E-01
PTEN	10q23.31	89622870	del	3.7	4.9	8.08E-01
RB1	13q14.2	48877887	del	1.9	3.9	4.10E-01
CAPN3	15q15.1	42640301	del	0.0	0.0	NA
CDH1	16q22.1	68771128	del	1.9	1.0	8.72E-01
MAP2K4	17p12	11924141	del	4.3	5.9	7.07E-01

[a] : Amp : Focal amplification; Del : Homozygous deletion

[b] : Percentage of genomic alterations

[c] : Chi-squared test : CDK4i pretreated mBC *versus* CDK4i unpretreated mBC ; p-values in bold are significant

NA: Not Applicable. Frequencies at 0% in mBC population

Supplementary Table 11. Genomic regions with prognostic effect of copy number alterations in the not treated and treated by CDK4 inhibitor population before biopsies collection.

				Non pre-treated				Pre-treated			
				Univariate (OS)	*Multivariate (OS)*			*Univariate (OS)*	*Multivariate (OS)*		
Gene(s)	Chromosomal band	Location	Status[a]	P-value[b]	Overall survival (OS) HR (95% CI)	Raw p-value[b]	Corrected p-value	P-value[b]	Overall survival (OS) HR (95% CI)	Raw p-value[b]	Corrected p-value
**Candidate**											
ZMIZ1	10q22.3	80828792	Amp	0.84	1.2 [0.5 ; 2.8]	0.22	1	0.68	1.2 [0.3 ; 4.3]	0.96	1
FOXM1	12p13.33	2966847	Amp	0.11	3.0 [0.9 ; 9.6]	0.11	1	0.09	1.9 [0.5 ; 7.5]	**0.04**	0.5
**Discovery**											
AGR2	7p21.1	16831435	Amp	0.44	0.6 [0.1 ; 5.1]	0.51	1	0.09	0.2 [0.0 ; 1.7]	0.37	1
CPNE3 (MMP16)	8q21.3	87497059	Amp	0.66	0.8 [0.4 ; 1.8]	0.75	1	0.98	0.7 [0.2 ; 2.1]	0.73	1
CDK4	12q14.1	58141510	Amp	0.48	1.6 [0.5 ; 5.2]	0.71	1	0.51	1.5 [0.3 ; 7.1]	0.29	1
LGR5	12q21.1	71833550	Amp	0.82	0.9 [0.4 ; 2.0]	0.83	1	0.27	1.7 [0.5 ; 5.4]	0.51	1
NFKBIA	14q13.2	35870717	Amp	0.62	1.3 [0.5 ; 3.5]	0.68	1	0.43	0.5 [0.1 ; 2.0]	0.27	1
CCL1	17q12	32687347	Amp	0.72	0.9 [0.2 ; 4.0]	0.27	1	0.07	2.0 [0.7 ; 6.1]	0.19	1
KCNG1	20q13.13	49620193	Amp	0.87	0.9 [0.4 ; 1.8]	0.74	1	0.84	1.7 [0.5 ; 5.8]	0.83	1

[a] : Amp : Focal amplification

[b] : p-values in bold are significant

Supplementary Table 12. Genomic regions with prognostic effect of copy number alterations in the not treated and treated by CDK4 inhibitor in ER+/HER2- eBC population before biopsies collection.

				Univariate (OS)	*Multivariate (OS)*		
**Gene(s)**	**Chromosomal band**	**Location**	**Status[a]**	**P-value[b]**	**Overall survival (OS) HR (95% CI)**	**Raw p-value[b]**	**Corrected p-value**
**Candidate**							
ZMIZ1	10q22.3	80828792	Amp	0.68	0.8 [0.4 ; 1.6]	0.92	1
FOXM1	12p13.33	2966847	Amp	0.51	0.9 [0.5 ; 1.5]	0.97	1
**Discovery**							
NSL1 (ATF3)	1q32.3	212899495	Amp	**<0.01**	2.1 [1.2 ; 3.6]	0.55	1
AGR2	7p21.1	16831435	Amp	0.36	0.6 [0.1 ; 2.5]	0.49	1
CPNE3 (MMP16)	8q21.3	87497059	Amp	0.24	1.3 [0.8 ; 2.1]	0.33	1
CDK4	12q14.1	58141510	Amp	**<0.01**	2.0 [1.0 ; 4.2]	**0.03**	0.4
LGR5	12q21.1	71833550	Amp	0.07	1.4 [0.7 ; 2.5]	0.24	1
NFKBIA	14q13.2	35870717	Amp	1	1.0 [0.4 ; 2.1]	0.82	1
CCL1	17q12	32687347	Amp	0.88	0.7 [0.3 ; 1.9]	1.00	1
KCNG1	20q13.13	49620193	Amp	0.63	0.9 [0.6 ; 1.5]	0.05	0.71

[a] : Amp : Focal amplification; Del : Homozygous deletion

[b] : p-values in bold are significant
